# An unusual cause of left atrium compression by ultrasound: A case report

**DOI:** 10.1097/MD.0000000000042625

**Published:** 2025-08-08

**Authors:** Jiaqin Ren

**Affiliations:** a Department of Anesthesiology, The Affiliated Hospital of Guizhou Medical University, Guian Hospital, Guiyang, China.

**Keywords:** cirrhosis, Sengstaken–Blakemore tube, transthoracic echocardiography

## Abstract

**Rationale:**

Cirrhosis is a chronic liver disease that affects millions of people worldwide. Patients with cirrhosis are at increased risk of upper gastrointestinal bleeding, a life-threatening complication.

**Patient concerns:**

A 34-year-old male with cirrhosis was admitted to the emergency department with symptoms of vomiting fresh blood and dizziness. He developed extensive upper gastrointestinal bleeding and was treated with a Sengstaken–Blakemore tube (SBT), followed by unexplained sharp fluctuations in heart rate and blood pressure.

**Diagnoses:**

Transthoracic echocardiography revealed that the SBT tube had shifted and compressed the heart.

**Interventions:**

The SBT was extracted under an endoscope.

**Outcomes:**

After treatment, he received a good prognosis.

**Lessons:**

The SBT tube displacement was found through transthoracic echocardiography to avoid more serious complications ultrasound technology is a noninvasive and visual technology, which has great value in many disciplines.

## 1. Introduction

**Figure 1.**
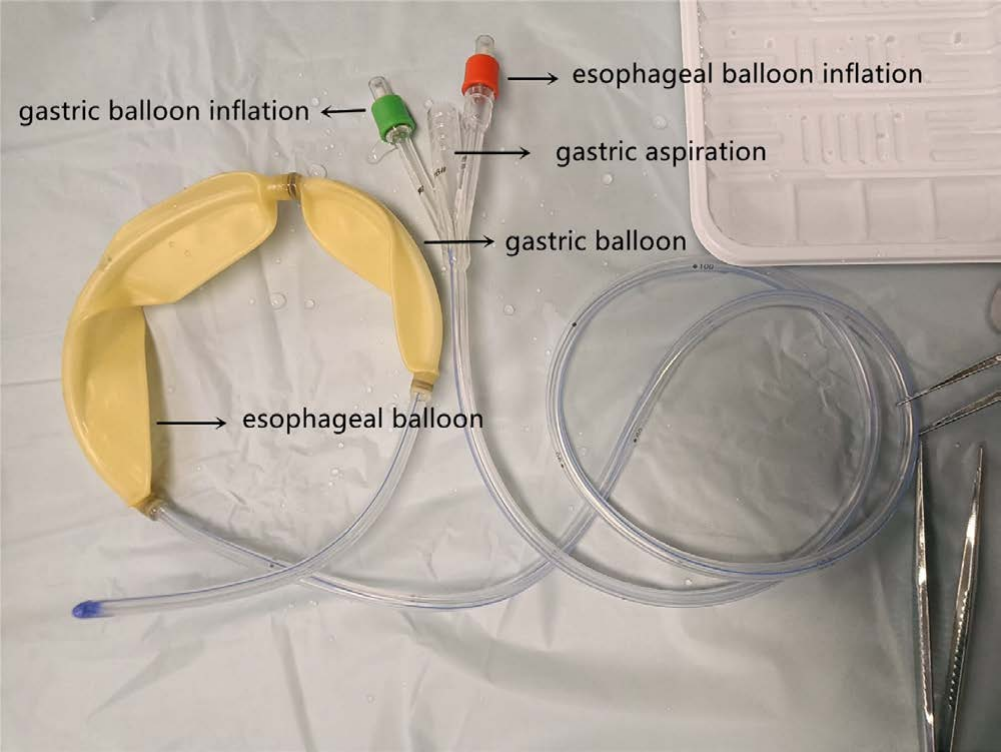
Schematic representation of Sengstaken-Blakemore tube composition.

The Sengstaken–Blakemore tube (SBT) has 2 balloons, one is called the gastric balloon, which is mainly placed in the fundus of the stomach to compress the gastric fundus vein by injecting gas through the green tube, The other is called the esophageal balloon, which is placed in the lower end of the esophagus to compress the esophageal vein by injecting gas through the orange tube. Another tube is a gastric suction tube, which is generally inserted into the stomach and used to pirate the contents of the stomach or inject medicine into the stomach (see Fig. 1 which demonstrates the schematic representation of SBT composition).

**Figure 2.**
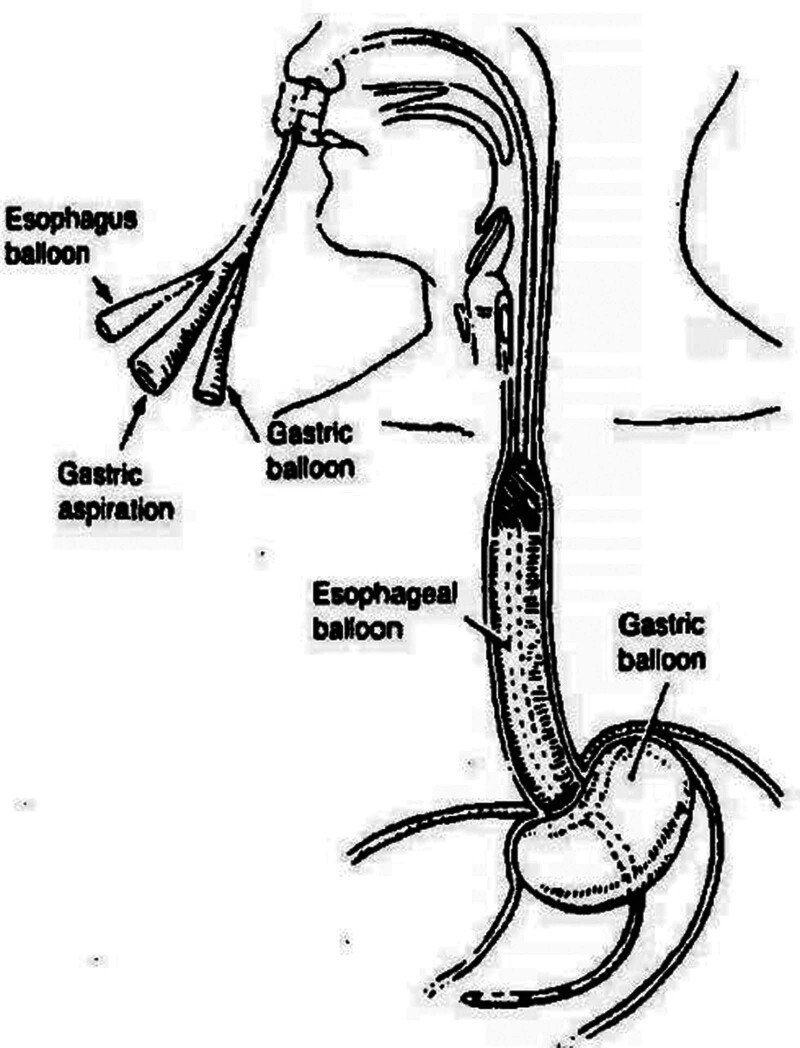
Schematic representation of Sengstaken-Blakemore tube placement. (Image courtesy from CR Bard, Inc.)

Upper gastrointestinal hemorrhage from gastric and esophageal varices rupture is a common and fatal complication in individuals with cirrhosis, with a mortality incidence ranging from 20% to 50%.^[1–3]^ Currently, endoscopic ligation is the recommended course of treatment.^[4]^ Even with exceptional ability, an endoscopist may not always be able to stop patients from bleeding uncontrolled. Still, with an 80% to 90% success rate in hemostasis, prompt SBT placement for compression hemostasis can save lives.^[5–7]^ The location of the SBT is often confirmed by auscultation or X-ray.^[8]^ The placement of SBT is shown in Figure 2 (see Fig. 2 which demonstrates schematic representation of SBT placement (Image courtesy from CR Bard, Inc.)). However, due to displacement, using SBT often leads to adverse events such as breathing obstruction, hypoxia, aspiration, perforation, rupture, and esophageal perforation in addition to airway injury.^[1,9–12]^ This case study describes a situation where an SBT was employed to treat upper gastrointestinal hemorrhage, and transthoracic echocardiography (TTE) was utilized to identify its displacement. Written informed consent was obtained for the publication of this case report.

## 2. Case description

A 34-year-old male patient was admitted to the emergency department with symptoms of vomiting fresh blood and dizziness. He had previously been diagnosed with hepatitis B cirrhosis with esophageal varices 2 years earlier but refused any treatment, and he had no other diseases. Following admission, the patient suddenly vomited 3000 mL of fresh red blood and lost consciousness with hemodynamic instability. Resuscitation procedures were immediately taken. To control the bleeding, an SBT was placed promptly, the location of the SBT was ascertained by listening for the sound of air over water in the stomach with a stethoscope when the air was inject into to the hollow tube, After filling the gastric balloon with 200 mL of air, the esophageal balloon with 60 mL of air, and 500 mL of normal saline was deployed to maintain traction, the bleeding was eventually stopped. The nostril’s scale was 45 cm.

**Figure 3.**
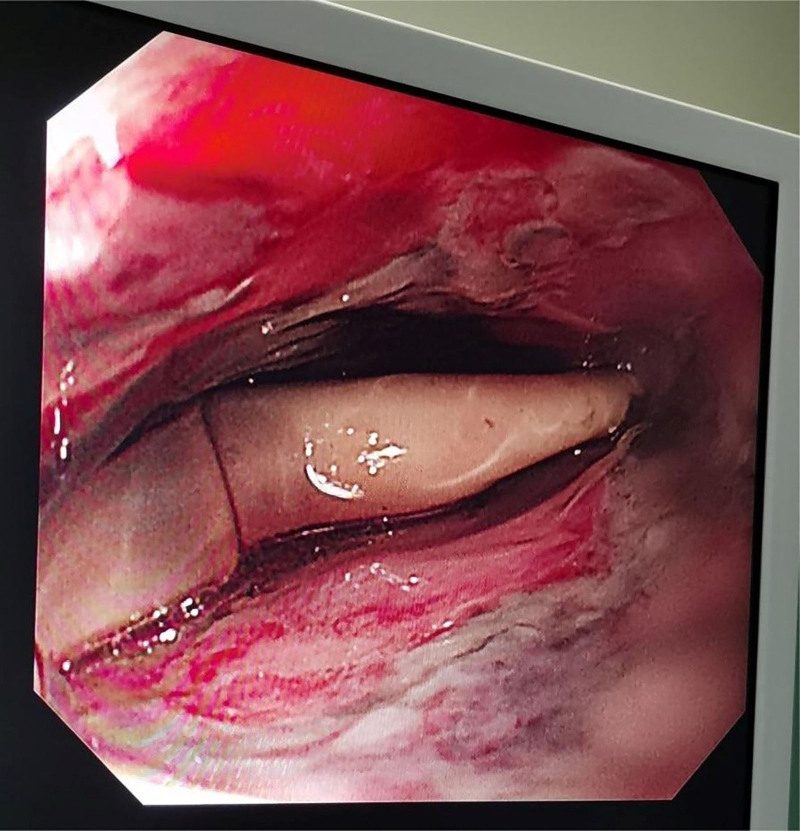
The displaced SBT on endoscopy, 2 balloons could not be correctly named, and severe edema and bleeding of the esophageal wall. SBT = Sengstaken–Blakemore tube.

To relieve mucosal compression, the balloons were deflated every 12 hours for 30 minutes, then reinflated with the same volume of air, and traction was reinstated. 4 days passed with no further bleeding until the morning of the 5th day, 1 hour after we tried to end SBT treatment, that was, after the balloons had been deflated, he suddenly vomited about 50 mL of fresh blood after a cough. Upon inflating the balloons at this time, the heart rate increased, whereas blood pressure decreased (HR:147 bpm, BP:87/47 mm Hg). Bedside point-of-care cardiac ultrasound was performed to determine the etiology of the hemodynamic instability, it revealed the left atrium (LA) being compressed by the balloon (see video S1, which demonstrates the LA had limited relaxation due to compression in the parasternal long axis; and see video S2, which demonstrates the LA is compressed with a significant reduction in volume, at the same time, the echogenic balloon wall can be seen in the apical 4-chamber), we immediately deflated balloons, the patient’s heart was no longer compressed and the heart rate dropped to normal and blood pressure stabilized (HR:87 bpm, BP:136/67 mm Hg). The SBT was still 45 cm away from the nostril display, after 2 hours of observation, the patient was hemodynamically stable and did not bleed again. We decided to withdraw the SBT, but we failed. Subsequent bedside endoscopy surprisingly revealed 2 sacculus of SBT visible 30 cm from the lip (see Fig. 3 which demonstrates the displaced SBT. On endoscopy, 2 balloons could not be correctly named, and severe edema and bleeding of the esophageal wall). Under endoscopy, 2 balloons were partially entangled, and were in a left and right position in the esophagus. The endoscopist skillfully and carefully dissected the SBT slowly and without bleeding.

After treatment, his condition was stable for more than a month, and he underwent a barium meal examination of the digestive tract, which showed an expected esophageal stenosis. And then he was treated with esophageal dilatation and received a good prognosis.

## 3. Discussion

SBT is commonly used for upper gastrointestinal bleeding, but can also be used in lower gastrointestinal bleeding, obstetric, and chest patients.^[13–15]^ Balloon compression of ruptured esophagogastric fund esophageal veins to reduce bleeding was used as early as the 1950s,^[16]^ the success rate of hemostasis can reach 80% to 90%.^[5–7]^ SBT is usually placed through the nostril by experienced caregivers when bleeding from the esophagogastric fundus vein is difficult to control in patients with liver disease, and as a transitional treatment option to buy enough time for other treatments.^[5,17]^ Air is then pushed into the gastric suction tube, and a stethoscope is used to detect the sound of bubbles or an X-ray is used to confirm that the tip of the tube, or an ultrasound can be used to locate the catheter tip in the stomach.^[8,18]^ An external traction about 500 g of pressure is then used and secure with tape. The gastric and esophageal balloons are usually kept inflated for 12 to 48 hours,^[8]^ and to prevent injury or necrosis. The common complications of using the SBT include chest infection, esophageal tear, tracheal obstruction, ulcer necrosis at the compression site, aspiration, asphyxia, perforation, esophageal perforation, aspiration pneumonia, acute cardiovascular disease, and rebleeding esophageal perforation.^[1,9–12]^

In this case, the placement of the SBT was initially determined by auscultation, and the hemostasis was effective until the 5th day, severe fluctuation in breathing and circulation occurred. TTE revealed an anomalous size of the LA, which is located anterior to the mid-esophagus (30 cm from the lip), and hemodynamic instability was generated by compression of the LA when the gastric balloon was shifted to inflate at 30 cm under endoscopy, despite the fact that the scale at the nostril was 45 cm. Endoscopic dissection revealed that the translocation of the gastric balloon to the esophagus resulted in the narrowing of the esophageal cavity, and due to edema and decay of the esophageal wall, the gastric balloon was embedded in the esophageal wall and tied with the esophageal balloon. This was why the scale did not show that the balloon has shifted.

Repositioning of the balloon during the use has never been reported in patients with SBT. We advocate the intermittent use of noninvasive ultrasonography during SBT use to ensure the location of the SBT. Especially for critically ill patients who cannot be moved. In this case, for example, we used the ultrasound technique not only to just check that the hollow tube of the SBT was in the stomach as in the past,^[18]^ but also to check that the balloon was not moving toward the cephalic end, especially after each inflation of the balloon. Moreover, this patient is not able to undergo a high frequency X-ray examination to determine the location of the SBT, which not only increases the rate of tube detachment but also increases the medical cost. In this case, the SBT tube displacement was found through TTE to avoid more serious complications. Ultrasound technology is a noninvasive and visual technology, which has great value in many disciplines.

## Author contributions

**Conceptualization:** Jiaqin Ren.

**Data curation:** Jiaqin Ren.

**Methodology:** Jiaqin Ren.

**Project administration:** Jiaqin Ren.

**Writing – original draft:** Jiaqin Ren.

**Writing – review & editing:** Jiaqin Ren.
